# Potential use of lymph node-derived HPV-specific T cells for adoptive cell therapy of cervical cancer

**DOI:** 10.1007/s00262-016-1892-8

**Published:** 2016-09-12

**Authors:** Mariëtte I. E. van Poelgeest, Valeria V. Visconti, Zohara Aghai, Vanessa J. van Ham, Moniek Heusinkveld, Maarten L. Zandvliet, A. Rob P. M. Valentijn, Renske Goedemans, Caroline E. van der Minne, Els M. E. Verdegaal, J. Baptist M. Z. Trimbos, Sjoerd H. van der Burg, Marij J. P. Welters

**Affiliations:** 1 Department of Gynecology, Leiden University Medical Center, Leiden, The Netherlands; 2 Department of Medical Oncology, Leiden University Medical Center, Building 1, K1-P, PO box 9600, 2300 RC Leiden, The Netherlands; 3 Clinical Pharmacy and Toxicology, Leiden University Medical Center, Leiden, The Netherlands; 4 Department of Microbiology Diagnostic, University of Rome “La Sapienza”, Rome, Italy; 5 Department of Medical Microbiology, Free University Medical Center, Amsterdam, The Netherlands

**Keywords:** Human papillomavirus, Tumor-draining lymph node, Cervical cancer, Adoptive cell transfer, HPV-specific T cells, Good manufacturing practice

## Abstract

Adoptive transfer of tumor-specific T cells, expanded from tumor-infiltrating lymphocytes or from peripheral blood, is a promising immunotherapeutic approach for the treatment of cancer. Here, we studied whether the tumor-draining lymph nodes (TDLN) of patients with human papillomavirus (HPV)-induced cervical cancer can be used as a source for ACT. The objectives were to isolate lymph node mononuclear cells (LNMC) from TDLN and optimally expand HPV-specific CD4+ and CD8+ T cells under clinical grade conditions. TDLN were isolated from 11 patients with early-stage cervical cancer during radical surgery. Isolated lymphocytes were expanded in the presence of HPV16 E6 and E7 clinical grade synthetic long peptides and IL-2 for 22 days and then analyzed for HPV16 specificity by proliferation assay, multiparameter flow cytometry and cytokine analysis as well as for CD25 and FoxP3 expression. Stimulation of LNMC resulted in expansion of polyclonal HPV-specific T cells in all patients. On average a 36-fold expansion of a CD4+ and/or CD8+ HPV16-specific T cell population was observed, which maintained its capacity for secondary expansion. The T helper type 1 cytokine IFNγ was produced in all cell cultures and in some cases also the Th2 cytokines IL-10 and IL-5. The procedure was highly reproducible, as evidenced by complete repeats of the stimulation procedures under research and under full good manufacturing practice conditions. In conclusion, TDLN represent a rich source of polyclonal HPV16 E6- and E7-specific T cells, which can be expanded under clinical grade conditions for adoptive immunotherapy in patients with cervical cancer.

**Electronic supplementary material:**

The online version of this article (doi:10.1007/s00262-016-1892-8) contains supplementary material, which is available to authorized users.

## Introduction

Cancer of the cervix uteri is the fourth most common cancer among women worldwide, with over 500,000 new cases and 200,000 deaths each year [1]. It is induced by persistent infection with oncogenic types of HPV, of which high-risk type 16 accounts for over 50 % of the cases [2]. Whereas early-stage cervical cancer displays a low recurrence risk after treatment (15 %), this is up to 70 % for more advanced stages [3–6]. Metastatic cervical cancer is prototypically chemotherapy resistant and requires better treatments.

Adoptive transfer of tumor-specific T cells is a promising immunotherapeutic treatment modality for patients with advanced or recurrent cancer. Infusions of ex vivo expanded TILs resulted in tumor regressions of lymphoma, Hodgkin’s disease and melanoma [7–9]. Good clinical responses following ACT are related to recognition of tumor-specific antigens [10, 11] by both CD4+ and CD8+ T cells. Since (metastatic) cervical cancer cells express the viral E6 and E7 oncoproteins, these are good tumor-specific antigens to be targeted by ACT. Indeed, strong spontaneous and vaccine-induced T cell responses to HPV16 E6 and E7 are protective and able to induce regression of premalignant lesions [12–14], whereas ACT with TIL containing HPV-specific T cells of metastatic cervical cancer patients resulted in an objective clinical response in three of the nine treated patients [15].

Our studies on the systemic and local HPV16-specific T cell response in cervical cancer patients revealed that these responses are detectable in only 30 % of the blood [16] and 35 % of the tumor specimens [17] of these patients, indicating that blood or tumor tissue may not function as a consistent source for HPV-specific T cells. However, the TDLN of patients with cervical cancer more frequently (50–83 %) contain HPV-specific T cells [17, 18] reactive to many epitopes [18], suggesting that TDLN may form better starting material to obtain cells for ACT. To investigate this, we expanded and characterized HPV-specific CD4+ and CD8+ T cells from lymph node mononuclear cells (LNMC) in patients with HPV16-induced cervical cancer under clinical grade (i.e., GMP) conditions and found that this reproducibly led to the expansion of polyclonal HPV16-specific type 1 T cell populations.

## Materials and methods

### Subjects

Eleven women with histologically proven early-stage HPV16-positive cervical cancer enrolled in the CIRCLE study, which investigates cellular immunity against HPV16+ anogenital lesions, were analyzed for the presence of HPV16+-specific T cells in their TDLN. HPV status was tested using HPV16-specific primers on DNA isolated from surgical resection specimens [19]. The patients were staged according to International Federation of Gynecology and Obstetrics (FIGO) and treated by radical hysterectomy and bilateral pelvic lymphadenectomy. The mean age was 44 years (median 42; range 28–65 years). Nine patients were staged FIGO IB1, one patient FIGO IB2 (C809) and one patient FIGO IIA (C800); all patients had squamous cell carcinoma. An additional 8 HPV16+ cervical cancer patients were incorporated of whom the blood samples were analyzed. Six patients were staged FIGO IB1, one patient IIA (C417) and one patient IA1 (C436). The study design was approved by the Medical Ethical Committee of the Leiden University Medical Center (LUMC), Leiden, The Netherlands.

### Isolation of peripheral blood mononuclear cells

Venous blood samples (54 mL) were drawn, and peripheral blood mononuclear cells (PBMC) were isolated using Ficoll density gradient centrifugation and cryopreserved as described earlier [14].

### Isolation and stimulation of lymph node-derived T cells

TDLN, suspected for tumor cell infiltration, were derived from the pelvic region and sampled during standard lymphadenectomy. The lymph nodes were cut into pieces, incubated for 1 h with collagenase IV (200 IU/ml, Sigma, St Louis, USA) and DNAse (50 mg/ml, Sigma) at 37 °C, after which a single cell suspension of the lymph node mononuclear cells (LNMC) was made using a cell strainer (BD, Erebodemgem, Belgium). LNMC samples were cryopreserved in 90 % FCS (Life Technologies, Bleiswijk, The Netherlands)/10 % DMSO (WAK-Chemie Medical, Steinbach, Germany) using a freezing machine and stored in the vapor phase of the liquid nitrogen. Alternatively, the lymph node was cut in half, and one side was scraped with a scalpel to obtain a layer of single cells of the LNMC, which were then cryopreserved. Only in two cases, patient C727 and patient C796, the resected lymph node used for the study was found to be tumor cell negative upon pathological examination.

For the test, LNMC were thawed, washed and seeded at a density of 0.5–2 × 10^6^ cells per well of a 24-well plate in 1 ml of IMDM (Lonza, Breda, The Netherlands) enriched with 10 % pooled human AB serum (Sanquin Bloodbank, Dordrecht, The Netherlands), penicillin (100 U/ml), streptomycin (100 µg/ml) and 2 mM l-glutamin (all from Lonza) and stimulated with HPV16 E6 and E7 clinical grade long peptide pools (5 µg/ml) in the presence of either recombinant human IL-2 (Aldesleukin, Novartis, Arnhem, The Netherlands) at 3 different concentrations (75, 150 and 300 U/ml) or a combination of 10 % T cell growth factor (TCGF; Zeptometric, Buffalo, USA) and 5 ng/ml recombinant human IL-15 (PeproTech, Hamburg, Germany), for a total of 22 days. The IL-2 and TCGF/IL-15 were added when culture medium was refreshed. Based on the results obtained, following LNMC were stimulated with GMP-grade materials only (C331, C427, C711-II, C707, C726-I and C726-II, and C727-I and C727-II), in which 150 U/ml IL-2 was added three times a week. In order to test the secondary expansion capacity [20, 21], 22-day cultured LNMC from C331, C427, C711 and C707 were re-stimulated using antigen-loaded autologous monocytes for another 3 weeks and then tested for responsiveness by proliferation, cytokine production and multiparameter flow cytometry. The expansion capacity was expressed as the fold change, which is the total number of viable cells at the end of the culture period (day 22) divided by the starting number of viable cells (both in case of the first and second expansion period). The remaining LNMC bulk culture cells were cryopreserved in 90 % FCS/10 % DMSO.

### Antigens

To stimulate LNMC, clinical grade HPV16 E6 and E7 long (25–35-mer) overlapping peptides (HPV16 E6: 9 peptides, HPV16 E7: 4 peptides) were synthesized in the GMP facility of the LUMC [13, 22]. To analyze the response of LNMC, a set of overlapping 22-mer peptides spanning the entire HPV16 E6 and E7 amino acid sequence as well as recombinant E6 and E7 proteins was synthesized [23]. The peptides were dissolved as described earlier [24].

### Analysis of T cell specificity and cytokine secretion

Expanded LNMC (50,000 cells/well) were stimulated with HPV16 E6 and E7 (22-mer) peptides (5 µg/ml) or protein (10 µg/ml)-pulsed autologous APC (i.e., B-LCL or monocytes) in triplicate wells in a 3-day proliferation assay. Medium only was taken along as a negative control, and PHA (0.5 µg/ml final concentration; HA16, Murex BioTech, Kent, UK) served as a positive control. After 48 h, 50 µl supernatant of each of the triplicate wells was pooled and stored at −20 °C. During the last 16 h of culture, 50 µl of 10 µCi/ml [^3^H]-thymidine (0.5 µCi/well; PerkinElmer, Groningen, The Netherlands) was added to measure proliferation as counts per minute (CPM) [24]. Stimulation indexes (SI) above 3, calculated as the average CPM of the triple-stimulated cell conditions divided by the average CPM of the three control wells consisting of LNMC with unloaded APC, were considered to be positive responses.

Antigen-specific production of cytokines in the supernatants of the proliferation assays was measured by ELISA (IFNγ and IL-10, according to the manufacturer Sanquin, Amsterdam, The Netherlands) and/or by the human Th1/Th2 cytometric bead array (BD Pharmingen) [14, 23]. Antigen-specific cytokine production was defined by a cytokine concentration above the cutoff value of the test (as defined by the manufacturer) and at least twice the concentration of the medium control (unloaded APC). The phenotype and poly-functionality of the T cell reactivity was examined by multiparameter flow cytometry as described previously [18]. Non-stimulated and PHA-stimulated LNMC served as negative and positive control, respectively. Responses were considered positive when the percentage of HPV-stimulated activated (CD154+ and/or CD137+) T cells and/or the percentage of cytokine producing (IL-2 and/or IFNγ) T cells was at least 3 times the medium control.

### Isolation of T cell clones

T cell clones from the TDLN of patient C331 were isolated using limited dilution as described earlier [17]. Briefly, CD4+ and CD8+ T cells were isolated using Dynabeads (Invitrogen, Carlsbad, CA, USA) or magnetic cell sorting (MACS; and detaching beads, Miltenyi Biotec, Bergisch Gladbach, Germany), respectively. Then, the cells were plated at 0.3 cells/well in IMDM (with 5 % human AB serum, 5 % FCS and 100 U/ml IL-2) and cultured in the presence of irradiated and overnight with HPV16 E6/E7 peptide-loaded autologous B-LCL as well as feeder cells (pooled PBMCs). Re-stimulation was done every 10 (CD8+) or 14 days (CD4+), and fresh medium with IL-2 was added weekly. The phenotype and TCRVβ usage [18] of T cell clones were tested by flow cytometry. Antigen specificity was determined by proliferation and cytokine production.

### Phenotyping of bulk cultures

The LNMC bulk cultures at day 22 were phenotyped by using three sets of markers and analyzed by flow cytometry. The cultured T cells were screened for the presence of regulatory T cells as described previously [23], the memory subtypes [25] and expression of inhibitory markers [26]. The memory set contains CD3-Pacific Blue (Clone UCHT1; DAKO), CD4-PE-CF594 (Clone RPA-T4; BD), CD8-APC-Cy7 (Clone SK1, BD), CD25-Brilliant Violet 605 (Clone 2A3, BD), CD27-Horizon V500 (Clone M-T271, BD), CD28-FITC (Clone CD28.2, BD), CD45RA-PerCP-Cy5.5 (Clone HI100, Biolegend), CD45RO-PE (Clone UCHL1, BD), CD62L-Alexa Fluor 700 (Clone DREG-56, Biolegend), CD95-PE-Cy7 (Clone DX2, Biolegend) and CCR7-Alexa Fluor 647 (Clone 3D12, BD). The inhibitory set consists of the same antibodies for CD3, CD4 and CD8 and additionally CD152-PE-Cy5 (Clone BN13, BD), CD279-Brilliant Violet 605 (Clone EH12.2H7, Biolegend) and TIM3-PE (Clone F38-2E2, Biolegend). The cryopreserved bulk LNMC were thawed, and after dead cell staining (yellow fixable live/dead staining kit, Invitrogen) for 20 min at room temperature (RT) in the dark, the cells (2 × 10^6^) were washed in PBS supplemented with 0.5 % bovine serum albumin (BSA), incubated for 10 min at RT in PBS/0.5 % BSA/10 %FCS (in the dark) to prevent nonspecific antibody binding and centrifuged, and the cell pellet was re-suspended in the antibody mixtures as described above and incubated for 20 min on ice (in the dark). Then, the cells were washed twice with PBS/0.5 % BSA and finally re-suspended in 1 % paraformaldehyde (LUMC). The acquisition on the Fortessa (BD) was performed immediately after the staining was finished. Analysis was performed by using DIVA software (version 6.2). An example of the gating strategy for the memory and inhibitory panel is provided in Supplementary Fig. 1. Following the frequency analysis, the data were subjected to a Multi-experimental viewer 4 (MeV4.0) software containing an algorithm for hierarchical clustering using Euclidean distance and complete linkage.

## Results

### HPV-specific primary and secondary expansion of TDLN-derived T cells

In a first attempt to expand HPV-specific T cells from TDLN, isolated LNMC of three patients (C394, C462 and C711, indicated by -I) were stimulated with antigen and cultured in the presence of different concentrations of IL-2 (75, 150 or 300 U/ml) or TCGF/IL-15, the latter based on our successful homeostatic culture protocol for TIL [17]. Cytokines were provided at the start, medium refreshment and split of the cultures. The expansion with 150 and 300 U/ml IL-2 was higher compared to 75 U/ml IL-2 or TCGF/IL-15 (Supplementary Fig. 2). There were no overt differences in the percentage of HPV-specific T cells between the cultures (Supplementary Fig. 3). Since this concentration of IL-2 was also used in our GMP-compliant ACT protocol for the generation of melanoma-specific T cell batches [27], the subsequent expansions of HPV-specific T cells from patient TDLN were performed with 150 U/ml of IL-2. LNMC were cultured from minced TDLN (C331, C427, C711, C707, C726, C727) and scraped TDLN (C800, C809, C796). After 22 days of culture, the expansion varied between fourfold and 92-fold (mean 36; median 29; Supplementary Fig. 4) and displayed exponential growth after 6–8 days (data not shown). Repeat with LNMC of the three initial patients (C711, C726, C727; indicated by -II or -III) showed a comparable expansion. Notably, the yield of T cells after 22 days of culture was not associated with the initial starting number of LNMC.

**Fig. 1:**
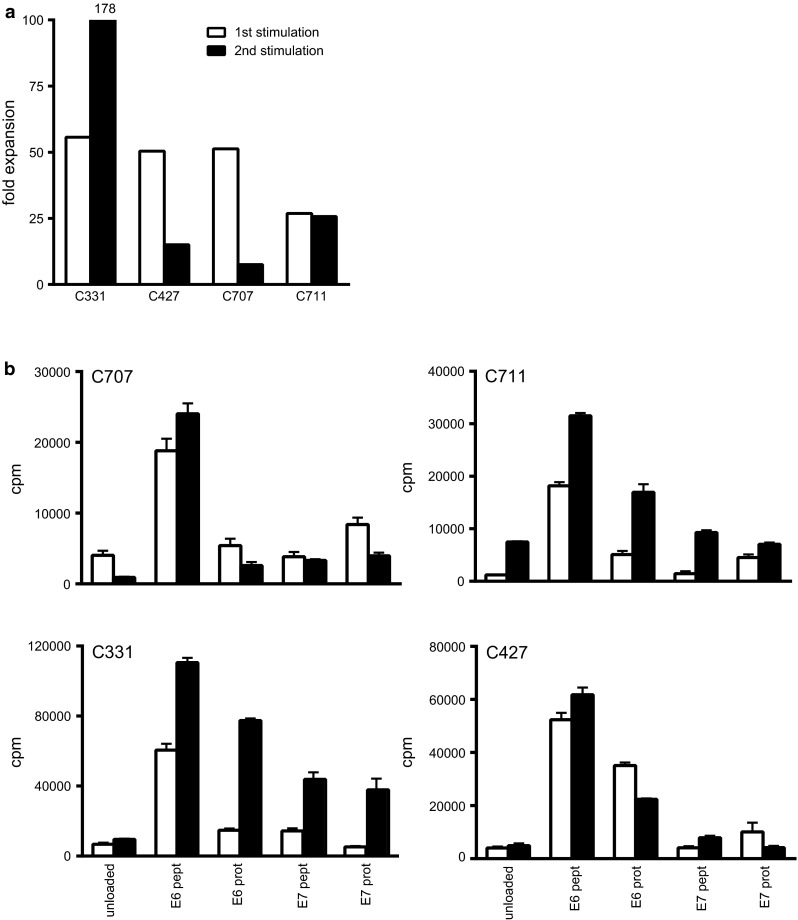
Expanded LNMC can be re-stimulated and remain specific and functional. LNMC cultures were prepared, and at day 22, these cells were subjected to another round of stimulation for again 3 weeks to determine whether the cells can be re-stimulated and do not express an exhausted phenotype. **a** The fold expansion of the first and second stimulation round is given as well as the proliferative response (mean plus standard deviation) as counts per minute (cpm; **b**), the latter being a measure for the incorporation of ^3^H-thymidine

To assess the peptide-specific secondary expansion capacity, the LNMC cultures of four patients (C331, C427, C707 and C711) obtained after the initial 22 days of stimulation were re-stimulated with HPV-specific E6 and E7 peptides loaded on autologous monocytes. All cultures expanded after this second stimulation, albeit that the fold expansion differed between patients and differed from the first expansion round (Fig. 1a). The strength of HPV-specific T cell proliferation (Fig. 1b) and cytokine production (Supplementary Fig. 5) was maintained after peptide-specific secondary expansion.

### Cytokine profiles of peptide-expanded HPV-specific LNMC

**Fig. 2:**
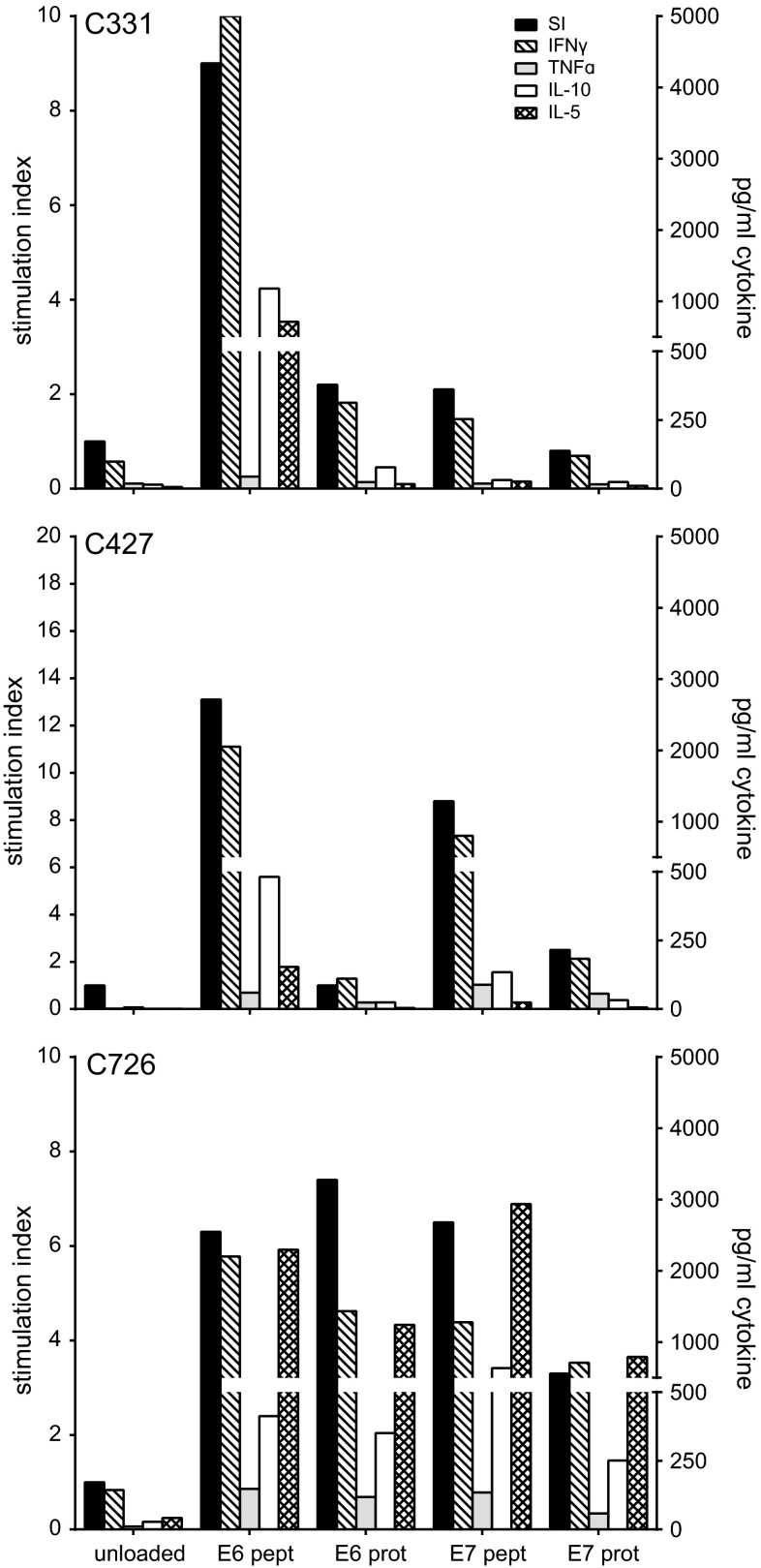
Representative examples of the proliferation and cytokine production of three LNMC cultures. The 22-day cultured LNMC were subjected to a 3-day proliferation assay to determine the response to HPV16 E6 and/or E7 22-mer peptides. Unloaded autologous monocytes served as negative control. The proliferative response is given as the stimulation index (SI) when stimulated with peptide- or protein-loaded autologous monocytes and is displayed on the *left y*-axis. The supernatant harvested after 48 h of stimulation was used for cytokine analysis by CBA, which is shown in the *right y*-axis

**Fig. 3:**
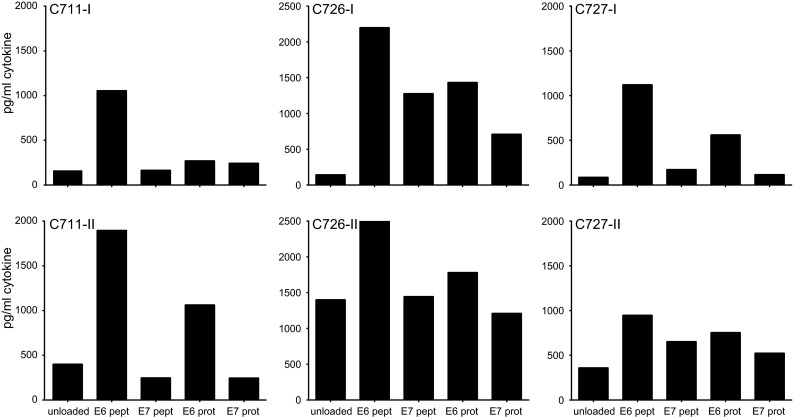
Complete repeat of LNMC cultures gives similar HPV16-specific responses. From 3 patients, the LNMC cultures could be repeated with the cryopreserved TDLN cells. The production of IFNγ upon stimulation with peptide- or protein-loaded autologous monocytes as measured by CBA is given. As a negative control, unloaded autologous monocytes are taken along. The results of both the first (-I) and second (-II) complete runs are displayed

The nine IL-2 expanded LNMC cultures and three repeated LNMC cultures (Supplementary Fig. 4) were analyzed for HPV specificity by their capacity to proliferate upon stimulation with HPV16 E6 and E7 peptides. Overall, HPV peptide-specific T cell proliferation was detected in the LNMC cultures of eight patients. HPV-specific IFNγ production was detected in all twelve tested cultures stimulated with peptide and in ten of the twelve LNMC cultures stimulated with protein (range 28 to >8000 pg/ml). In eleven cultures, also IL-10 and/or IL-5 was detected. Figure 2 shows three representative examples of HPV-specific proliferation and cytokine production. The cytokine profile varied from a predominant Th1 type (IFNγ and TNFα) to a mixed Th1/Th2 type (IFNγ, IL-10 and IL-5). The results of the three repeated cultures showed HPV-specific IFNγ production in two cases (Fig. 3). In the other case (C726-II), the LNMC were still too activated, indicated by the high level of IFNγ in the non-stimulated (unloaded) condition, but when analyzed by flow cytometry it did show HPV-specific reactivity. Thus, overall the stimulation procedure was reproducible.

### Peptide-expanded LNMC consist of polyclonal HPV-specific CD4+ and CD8+ T cell populations

**Fig. 4:**
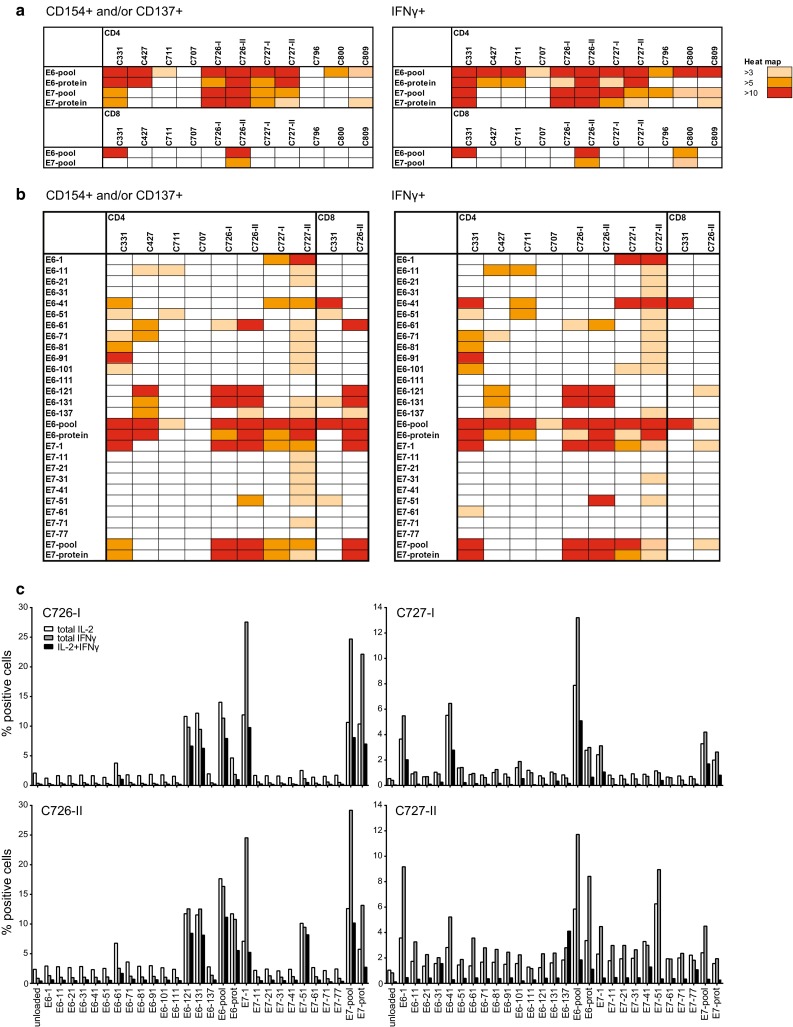
LNMC cultures harbor a broad HPV-specific T cell repertoire. The type and breadth of the HPV-specific T cell response in LNMC cultures were determined by intracellular cytokine staining in which both the activation and functionality of the T cells can be measured. LNMC were stimulated overnight with autologous monocytes loaded with peptide pools (**a**), single peptides or the corresponding whole protein (**b**) as indicated. The expression of the activation markers CD154 and CD137 were measured as well as the production of the cytokine IFNγ. Unloaded autologous monocytes are used as background staining. Depicted here is a heat map showing the fold induction above background staining for both the CD4+ and CD8+ T cells. A fold induction of at least three is considered positive. The results of a complete repeat of LNMC culture for two patients are included. Notably, from C796, C800 and C809, no single peptide analysis was performed. **c** Similar to **b**, the frequencies of the cytokine producing HPV-specific T cells are given for the LNMC cultures of the two patients that could be completely repeated. The results of both the first (-I) and second (-II) runs are given

To analyze the type and fine specificity of T cells responding to HPV, a multiparameter flow cytometric analysis was performed on the nine LNMC cultures stimulated with 150 U/ml of IL-2 cryopreserved at day 22. The percentage of CD4+ and CD8+ T cells expressing the activation markers CD137 (for CD4+ or CD8+ T cells) and/or CD154 (for CD4+ T cells) after stimulation with HPV16 E6 and E7 peptide pools or protein was examined to type and enumerate the HPV-specific T cells within the LNMC cultures. In all LNMC cultures, a HPV16-specific CD4+ T cell response, associated with IFNγ production, was detected (Fig. 4a). In seven cases, peptide recognition was paralleled with reactivity against protein-pulsed APC. In three patients also, HPV16-specific CD8+ T cells were detected.

To study the breadth of the responding T cell population, the 22-day expanded LNMC cultures of six patients were stimulated with single overlapping peptides of HPV16 E6 and E7. In addition, two of the repeated LNMC cultures (C726-II and C727-II) were taken along. Overall, seven out of these eight LNMC cultures displayed HPV16 E6 responses and five of them displayed HPV16 E7 reactivity (Fig. 4b). In five of the six patients, the CD4+ T cells responded to three or more different peptides. HPV-specific CD8+ T cell reactivity was detected in two patients. The reactivity in the repeated LNMC cultures was comparable, if not broader, to the primary expanded cultures of these patients (Fig. 4c). Thus, the culture procedures followed were robust and led to comparable results when repeated.

We had sufficient material of the TDLN of patient C331 to confirm the breadth of the HPV-specific T cell response by using T cell clones. In total, six CD4+ and five CD8+ HPV-specific T cell clones were obtained (Supplementary Table 1) that proliferated and produced IFNγ after stimulation with peptides. The result showed that the CD8+ T cells responded to E6 peptides 41 and 131/137, whereas CD4+ clones reacted to E6 peptides 91 or 101 as well as to E7 peptide 1 or 71 (Supplementary Table 1).

### Expansion of HPV-specific T cells from TDLN under full GMP conditions

**Fig. 5:**
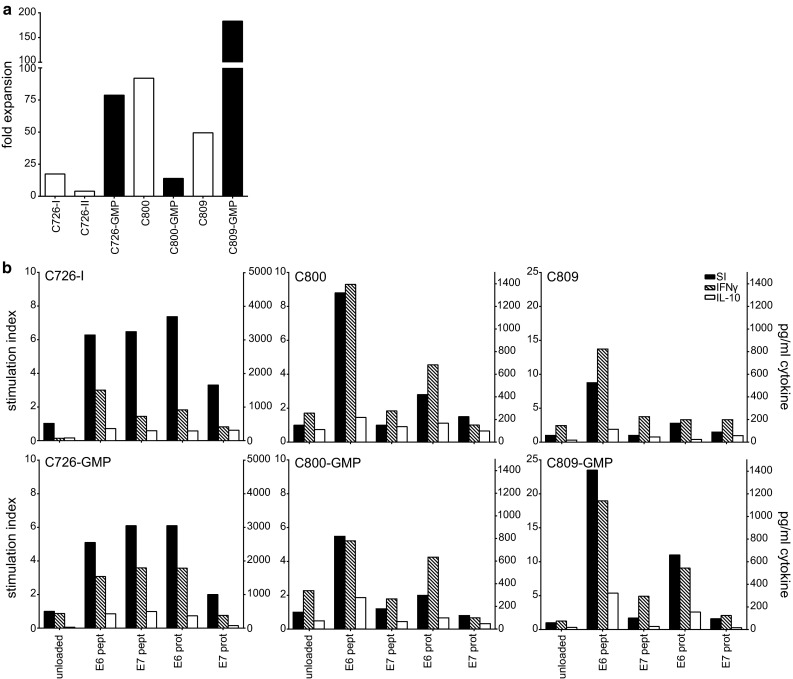
LNMC cultures can be performed successfully under GMP conditions. From 3 patients, the LNMC cultures were initiated under research conditions or full GMP conditions at the GMP facility of the LUMC. **a** The fold expansion of under both research (*white bars*) and GMP conditions (*black bars*) is shown. **b** The proliferation (indicated by stimulation index on *left y*-axis) and cytokine production by ELISA (indicated on *right y*-axis) in both LNMC cultures of the 3 patients are shown

To validate our method for clinical use, we completely repeated the protocol under full GMP conditions. TDLN of three patients (C726, C800 and C809) were stimulated and analyzed. HPV reactivity was assessed by IFNγ ELISA because this assay is validated by the manufacturer and specific IFNγ secretion is a release criterium for clinical T cell batches [27]. The expansion was 14- to 183-fold after 22 days of culture (Fig. 5a). In two patients (C726 and C809), the expansion was much better than previously observed, whereas in one case (C800), it was lower. Importantly, the HPV16-specific proliferation and cytokine production (IFNγ/IL-10) were comparable between the T cell batches cultured under research or full GMP conditions (Fig. 5b).

### HPV-specific LNMC cultures display an effector memory phenotype

Further characterization of all fifteen obtained peptide-stimulated LNMC cultures (both research and GMP), derived from cells initially expanded with 150 U/ml of IL-2, was performed by flow cytometry using an antibody panel for memory and inhibitory markers as well as to detect HPV-specific CD4+ CD25+ FoxP3+ regulatory T cells, for which we previously showed that they can be present in patients with HPV16-induced cancer [18, 28]. However, we did not find an overt expansion of CD25hi-FoxP3+ T cells (mean 1.65 %, range 0.42–5.08 % at day 22 compared with the results at the start of the culture; mean 1.26, range 0.21–2.30 %).

All LNMC cultures contained more than 90 % viable CD3+ T cells, and while between patients, the content of CD4+ and CD8+ T cells varied, this was not the case between repeated LNMC cultures of individual patients (Supplementary Fig. 6a). Due to the peptide stimulation in vitro, almost all cells were CD45RO+. The majority displayed an effector memory phenotype, albeit that in some cases, small percentages of central memory stem cells (Tscm) could be detected. Also in a few cultures, CD8+ effector memory CD45RA+ cells (TEMRA) were observed. For the five cases in which one or more repeats of the LNMC stimulation procedure were performed (C711, C726, C727, C800 and C809), the repeats nicely clustered together per patient when hierarchical clustering was used (Supplementary Fig. 6b). The LNMC were also stained for the expression of inhibitory markers PD-1, TIM3 and CTLA-4. The majority of the CD4+ T cells expressed PD-1 often in combination with TIM3, whereas only a minority of the CD8+ T cells expressed PD-1 and/or TIM3. CTLA-4 expression was hardly detected on T cells (Supplementary Fig. 7a). Although the expression of these markers varied between the LNMC cultures of different patients, there were no overt differences in the repeated LNMC cultures from individual patients (Supplementary Fig. 7b). Altogether, the results show that the peptide-stimulated LNMC cultures are of an activated effector memory T cell phenotype. There was no relation between the expression of the different markers analyzed and the frequencies of HPV-specific T cells as determined by intracellular cytokine staining (Fig. 4).

### The potential use of PBMC as source for HPV-specific T cells

**Fig. 6:**
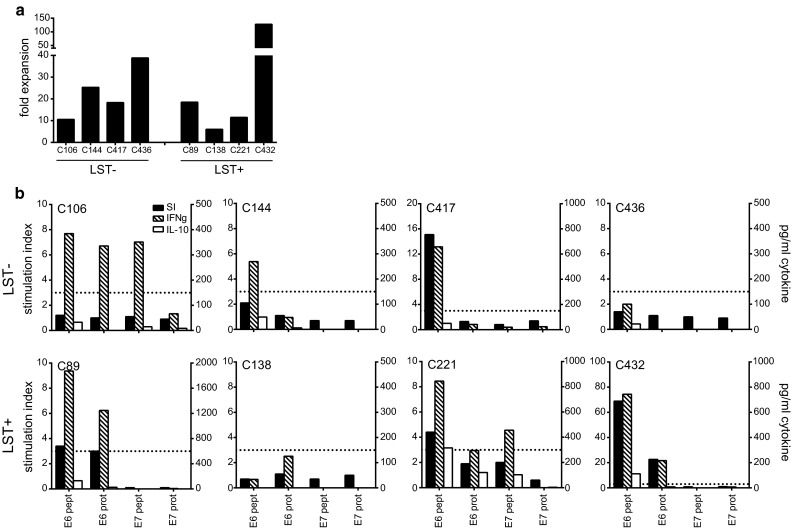
HPV-specific bulk cultures can be obtained from PBMCs of HPV16+ cervical cancer patients. PBMCs of HPV16+ cervical cancer patients were cultured according to the LNMC culture protocol and after 3 weeks subjected to the proliferation assay and cytokine analysis. Four patients with a previously negative response as determined by the lymphocyte stimulation test (LST−) and 4 patients with a positive reactivity (LST+) were selected for this purpose. **a** The fold expansion of the PBMC cultures are depicted. **b** The proliferative response (indicated by stimulation index (SI) on the *left y*-axis) and cytokine production as determined by ELISA (indicated on *right y*-axis) are given. The *dotted line* indicates a SI of 3, which is defined as the threshold for a positive proliferative response

The data of the directly ex vivo performed lymphocyte stimulation test (LST) with PBMCs of cervical cancer patients suggested that LNMC were more useful than PBMC as a source for HPV-specific T cells [16]. The LST is a proliferation assay in which freshly isolated PBMCs are stimulated with HPV peptides for a total of 7 days and proliferation is measured using ^3^H-thymidine. To validate this, we applied the HPV peptide stimulation protocol with 150 U/ml IL-2 as described above to blood samples, obtained before radical surgery, from eight HPV16+ cervical cancer patients. We selected four patients displaying a HPV-specific proliferative response in PBMC (LST+), and as control four patients without such response (LST−). The fold expansion (Fig. 6a) and HPV specificity (Fig. 6b) were determined. In three of the four LST+ patients, the expanded PBMC displayed a secondary proliferative response when stimulated with HPV antigens. In addition, also in one of the PBMC cultures of LST− patients, a secondary HPV-specific proliferation was found. Subsequently, we measured the production of IFNγ and IL-10 in the supernatant of these cultures. IFNγ was detected whenever the PBMCs displayed an HPV-specific proliferative response. Unexpectedly, HPV-specific IFNγ production was also detected in two of the PBMC cultures of LST− patients (C106 and C144) that did not show HPV-specific proliferation. The results demonstrate that although PBMCs from HPV16+ cervical cancer patients can be used for enriching and expanding HPV16-specific T cells, based on the secondary proliferative capacity of the stimulated PBMC, this will not be possible in all patients and the highest success rate will be obtained in those patients previously showing a positive LST response.

## Discussion

In this explorative study, we found that isolation and expansion of HPV16-specific LNMC derived from TDLN of patients with HPV16-induced cervical cancer are feasible and result in the generation of a polyclonal HPV-specific T cell response in all eleven tested patients. After stimulation with GMP-grade E6 and E7 peptides and IL-2, the LNMC expanded ~36-fold. The expansion of HPV16-specific CD4+ T cell was found in all nine patients tested in detail, and in three patients, also HPV16-specific CD8+ T cells were detected. The bias toward CD4+ T cell reactivity against HPV-derived epitopes is not likely a result of the culture method used here, but more a reflection of what is generally found in the spontaneous T cell response to HPV in cervical cancer [17, 18, 29–31], as well as among TILs from patients with head and neck cancer [32]. The T helper type 1 (Th1) cytokine IFNγ was produced in all LNMC cultures and in some cases also the Th2 cytokines IL-10 and IL-5. Importantly, the procedure was reproducible as complete repeats of the stimulation procedures under research and under full GMP conditions showed similar results when compared to the first runs. Promising results already have been obtained in a pilot study in colorectal cancer patients, as immunotherapy or in adjuvant setting, using TDLN-expanded T cells for ACT [33, 34].

The HPV peptide-stimulated LNMC cultures predominantly contained HPV16-specific CD4+ T cells, producing IFNγ and/or IL-5. HPV16-specific T cells with this mixed cytokine profile were also found in antigen-experienced healthy individuals [35] and in patients with a complete regression of their HPV16-induced high-grade vulvar lesion after therapeutic vaccination [13, 14, 23], indicating that the responding LNMC cells acquired an appropriate cytokine profile during the stimulation procedure. The outgrowth of tumor-specific T cells of only a CD4+ phenotype should not pose a problem for their use in ACT. Although successes have been achieved with ACT products containing merely tumor-directed CD8+ T cells [36, 37], there are indications that CD4+ T cells can help or can do the job. Substantial or complete tumor regressions have been achieved by ACT of T cells consisting only or mainly of CD4+ T cells [7, 38–40]. Transferred CD4+ T cells can contribute to antigen spreading [38], enhance the recruitment of CD8+ T cells to the tumor as well as sustain their effector function [41], reduce CD8+ T cell exhaustion [42], switch tumor-induced M2 macrophages to activated M1-like macrophages [43] and kill tumor cells via direct and indirect mechanisms [44, 45]. Thus, the infusion of tumor-specific Th1 cells may have great clinical benefit by altering the tumor micromilieu into a preferred type 1 cytokine-associated immune contexture [46].

In a number of ACT studies in metastatic melanoma, the tumor-specific T cells are derived from PBMC [27, 47, 48]. Based on our previous study on HPV-specific T cell responses in almost 100 patients with cervical cancer, we predicted that the PBMC of patients with cervical cancer would be an inferior source for HPV16-specific T cells than TDLN. We stimulated the PBMCs of eight different HPV16+ cervical cancer patients and detected a secondary proliferative response with production of IFNγ in four patients, three of which was expected based on the earlier conducted LST. The stimulated and expanded PBMC of two additional patients produced only IFNγ when stimulated with HPV16 antigens, suggesting that these cells lost their capacity of secondary peptide-specific expansion. These data suggest that PBMCs can be used as a source for successful expansion of HPV-specific T cells in about 50 % of the cases, and the highest success rate (100 %), however, is obtained when TDLN are used as source.

The relatively low numbers of obtained T cells after ex vivo expansion may be a limitation in ACT studies. Success rates of 50–70 % have been described in ACT studies in patients with metastatic melanoma [8, 37, 49]. In these studies, the functional properties, including the expansion rate and the presence of Th1 cytokines, and the total number of the transfused T cells were shown to correlate with clinical success. Most melanoma patients in the first ACT studies received over 10^11^ cells. In this study, we were able to expand 1–5 × 10^6^ LNMC to 36–180 × 10^6^ T cells per patient, respectively. It is not likely that the amount of starting material will be more in a clinical setting. Lymph node material will have to be collected during histological biopsy, and in our experience, on average 12 × 10^6^ (range 0–72 × 10^6^; median 5.6 × 10^6^; *n* = 48) LNMC are obtained when TDLN isolated during pelvic lymphadenectomy are scraped (unpublished data). There is evidence that ACT with lower numbers of T cells also results in clinical benefit. A recent study in melanoma patients showed that transfusions of on average 5 × 10^8^ T cells lead to clinical responses in half of the patients [27]. Moreover, a single transfusion of 0.37–2.2 × 10^5^ cytomegalovirus-specific CD8+ T cells/kg body weight after allogeneic stem cell transplantation leads to viral clearance in acute leukemia patients [50]. As the expanded numbers of LNMC using the current protocol will be lower than transferred to melanoma patients but the tumor antigens are known, there is the option to increase the effects of ACT by vaccination following T cell transfer [46]. We recently showed that vaccination induced in vivo massive (<1000-fold) clonal expansions and regression of tumors in a mouse melanoma model [51]. Notably, in HPV-driven cancers, the tumor-specific antigens are always the same. Hence, the use of an off-the-shelf vaccine, such as the HPV16 synthetic long peptide vaccine with proven strong immunogenicity in end-stage cervical carcinoma patients [22, 26, 52, 53], following ACT may lead to massive numbers of HPV-specific polyclonal T cells in patients with HPV16-induced cancer.

Taken together, our results show that all TDLN cell cultures isolated from patients with HPV16-induced cervical cancer are a rich source of polyclonal HPV-specific Th1 and Th2 cells, which after proper ex vivo stimulation can be expanded under full GMP conditions. These HPV-specific T cells may be used in future ACT studies in patients with metastatic HPV16-induced cancer.

## Electronic supplementary material

Below is the link to the electronic supplementary material.

Supplementary material 1 (PDF 2772 kb)

## Abbreviations

CPM: Counts per minute
FIGO: Federation of Gynecology and Obstetrics
LNMC: Lymph node mononuclear cells
LST: Lymphocyte stimulation test
LUMC: Leiden University Medical Center
SI: Stimulation index
TCGF: T cell growth factor
Th: T helper
