# Supplementary material for: Potential use of lymph node-derived HPV-specific T cells for adoptive cell therapy of cervical cancer
Source: Cancer Immunol Immunother. 2016 Sep 12;65(12):1451–63. doi: 10.1007/s00262-016-1892-8 (PMC5099359; doi:10.1007/s00262-016-1892-8)
Supplement: Supplementary file 1 — Supplementary material 1 (PDF 2772 kb) [file 262_2016_1892_MOESM1_ESM.pdf]

Supplementary Table 1: LNMC isolated from patient C331 contains CD4+ and CD8+ HPV-specific T cells

clone	T cell type[1]	specificity[2]	Vβ[3]	antigen specific cytokine production[4]				
				IFNγ	TNFα	IL-10	IL5	IL-2
2	CD8	E6 131+137	4	268	-	-	-	-
3	CD8	E6 41	NT	161	-	-	-	-
4	CD8	E6 137	NIK	118	-	-	-	-
6	CD8	E6 41	11	1811	1639	-	73	159
9	CD8	E6 41	NT	137	92	-	-	-
11	CD4	E7 71	NIK	2324	926	-	144	380
13	CD4	E6 91	14	1252	427	-	117	350
14	CD4	E6 101	13.1	179	-	-	867	-
16	CD4	E7 1	NT	1512	188	-	4884	95
19	CD4	E7 1	NIK	4417	715	4287	5000	510
20	CD4	E6 91	14	1500	401	-	-	-

[1]Phenotype measured by flow cytometry.

[2]First amino acid of the 22-mer overlapping peptide loaded on autologous B-LCL that was recognized

[3]Vβ usage of clone analyzed by flow cytometry. NT=not tested, NIK= not in kit. (Notably, 30% of Vβ is not found by antibodies present in this kit)

[4]Cytokine production (in pg/ml) after 48 hours of culture on B-LCL as measured by cytometric bead array. Only levels at least 3 times higher than unstimulated cells (background) are shown. -, means production less than 50 pg/ml or not 3 times higher than background.

a. Memory panel

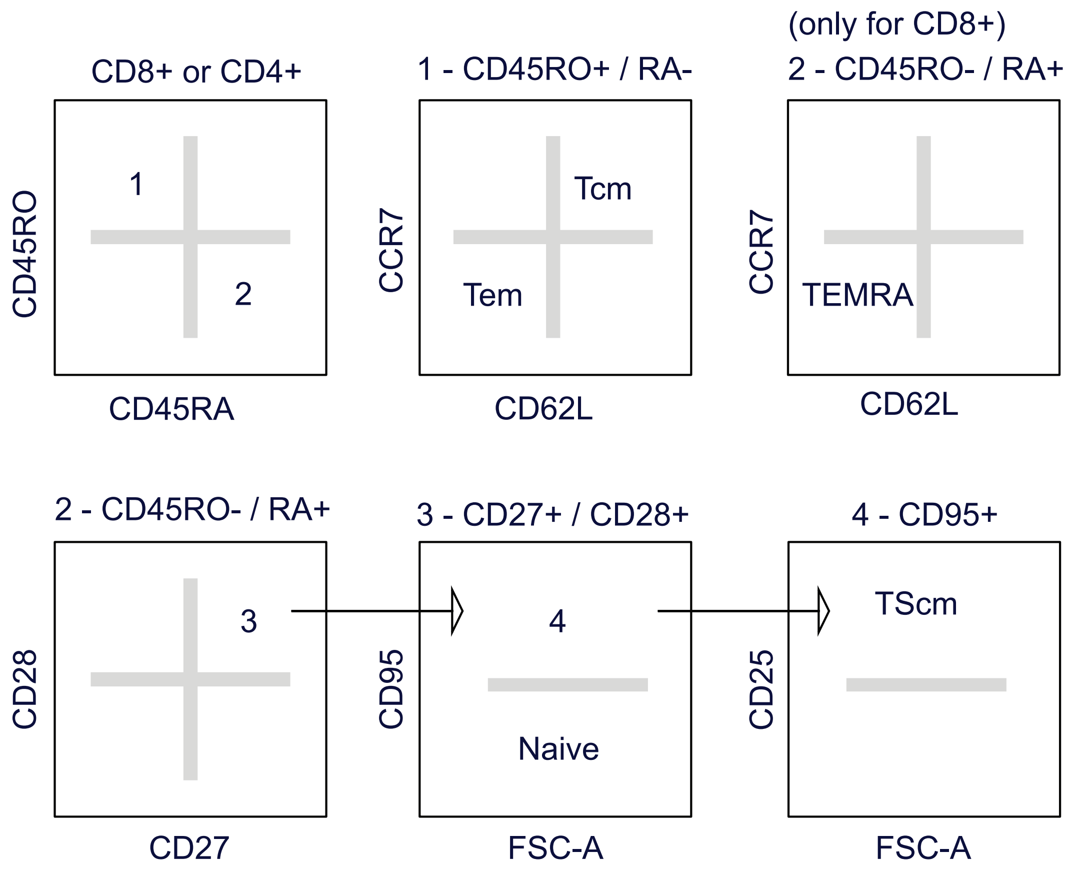

b. Inhibitory panel

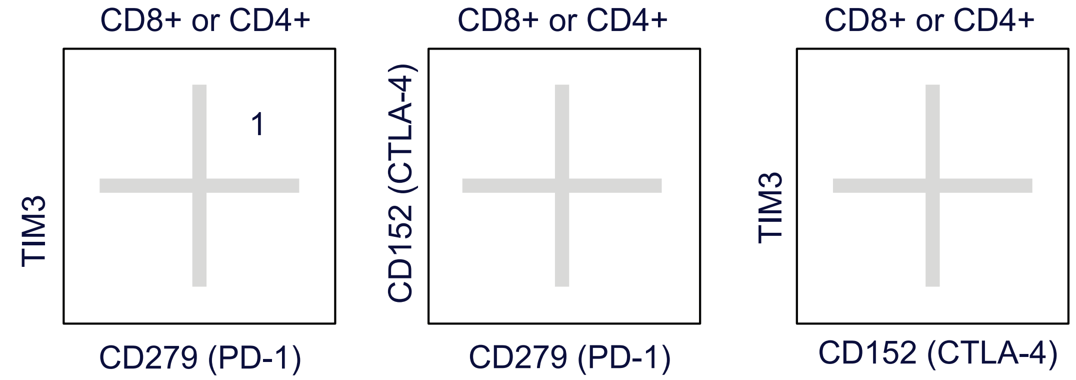

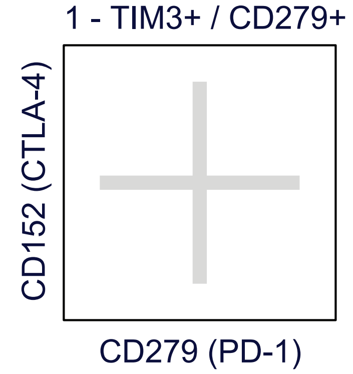

Supplementary Fig. 1 Gating strategy of memory and inhibitor panel. Upon selecting the singlet cells and the viable cells the CD3 cells were gated. Within these viable single CD3+ cells either the CD4+ or CD8+ cells were selected using a CD4 versus CD8 plot. **a** For the memory panel, subsequently, for each T cell type the CD45RA is plotted versus the CD45RO and the populations indicated by a 1 (CD45RO+/CD45RA-) and 2 (CD45RO-/CD45RA+) are depicted in a CD62L versus CCR7 plot to determine the frequency of effector memory T cells (Tem) within the population 1 of CD4+ cells, central memory T cells (Tcm) within the population 1 of both CD4+ and CD8+ cells and the terminally differentiated effector memory cells within population 2 of the CD8+ cells. In addition, the CD45RA+ CD4+ or CD8+ were plotted for CD27 versus CD28. The double positive cells (population 3) that were then CD95 positive (population 4) were displayed for forward scatter against CD25. The subsequent CD25 positive cells are the central memory stem cells (TScm). **b** For the inhibitor panel the pre-selection is again the same as for A and the CD4+ or CD8+ T cells are displayed for the different inhibitor marker combinations (TIM3 versus PD-1; CTLA-4 versus PD-1 and TIM3 versus CTLA-4). The double stained cells for TIM3 and PD-1 were also plotted for PD-1 versus CTLA-4 to determine the triple positive cell frequency.

a

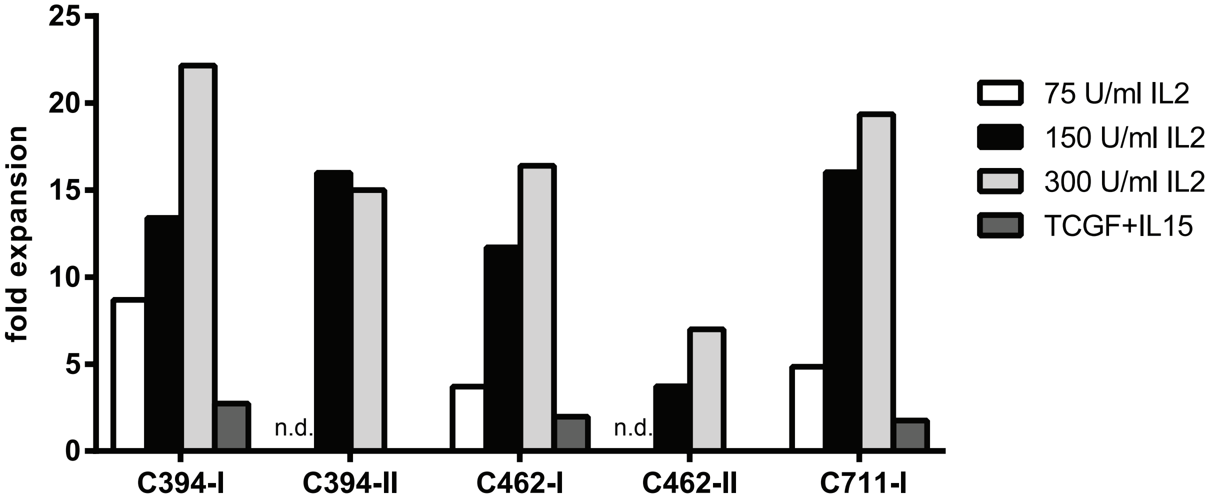

b

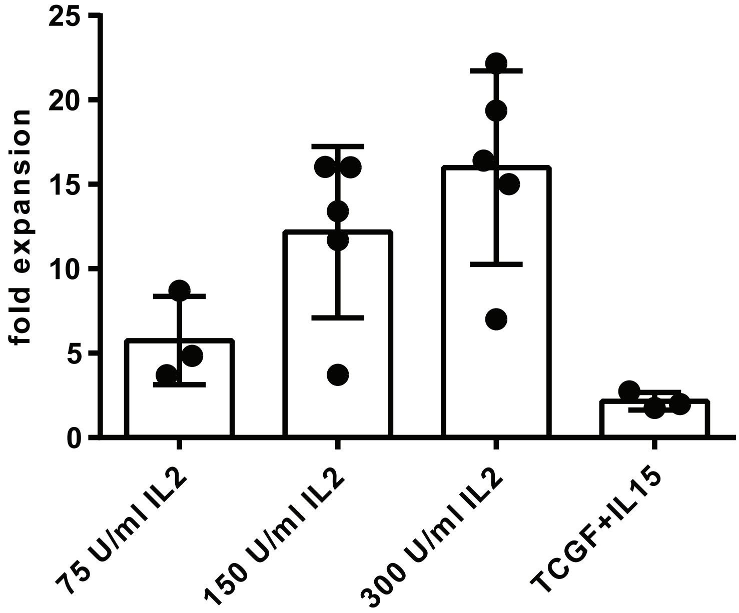

Supplementary Fig. 2 Expansion of LNMC under different conditions with IL-2 and TCGF/IL-15. LNMC cultures were first initiated using different concentrations of IL-2 (75, 150 or 300 U/ml) or the combination of TCGF and IL-15. The cytokines were provided ad libitum. **a** The fold expansion of LNMC cultures under different conditions is given. n.d. means not determined. **b** The fold expansion (mean plus standard deviation) is given when grouped according to the culture method.

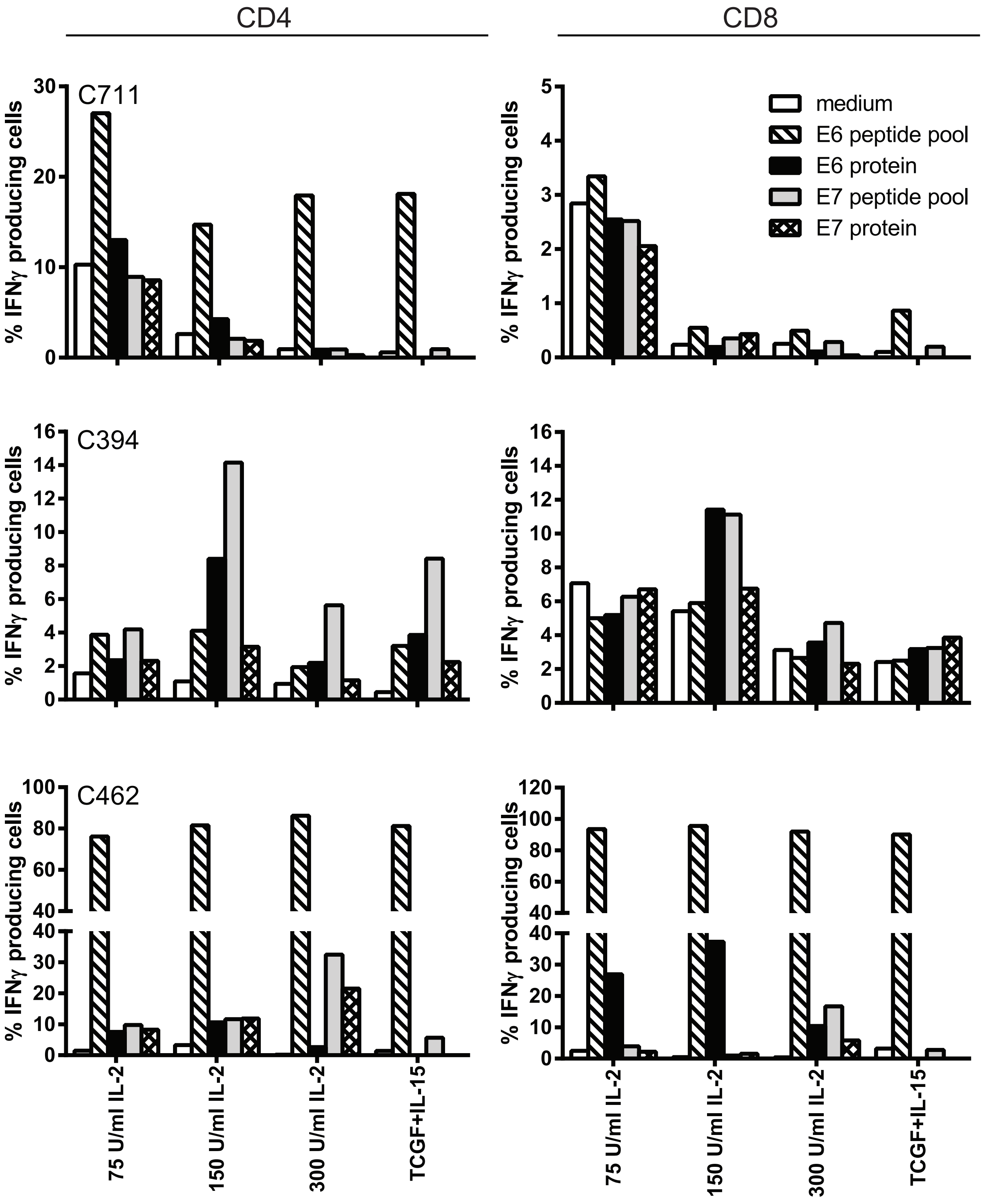

Supplementary Fig. 3 Expansion of LNMC under different conditions with IL-2 and TCGF/IL-15.
As in Supplementary Figure 2, LNMC were cultured with different concentrations of IL-2 (75, 150 or 300 U/ml) or the combination of TCGF and IL-15. The cytokines were provided ad libitum. An intracellular cytokine staining was performed on these cultured LNMC. The frequency of the IFNγ-producing cells are depicted when the cultured LNMC were stimulated with autologous monocytes loaded with the indicated peptide pools or protein of HPV16. Unloaded autologous monocytes served as negative control (here indicated by medium).

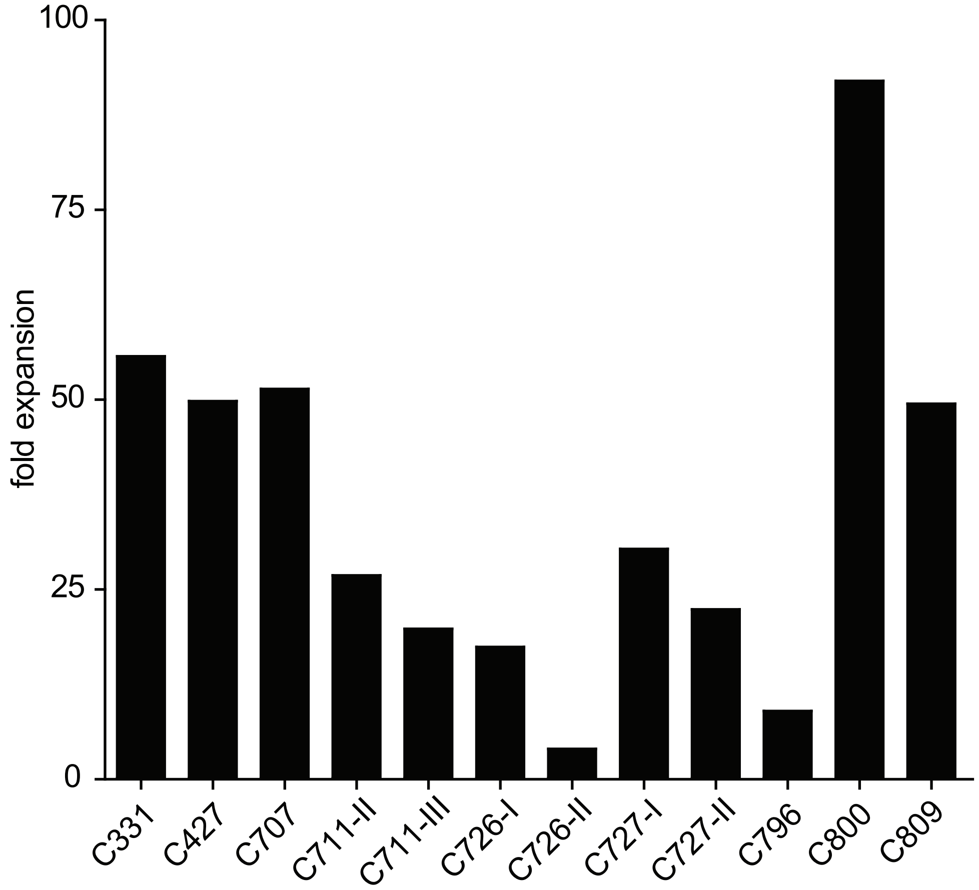

Supplementary Fig. 4 Expansion of the LNMC cultures stimulated with IL-2 every 2-3 days. LNMC cultures were refreshed every 2-3 days and/or divided over multiple wells with medium containing IL-2 to a final concentration of 150 U/ml. The fold expansion of the in this way obtained LNMC cultures are shown here.

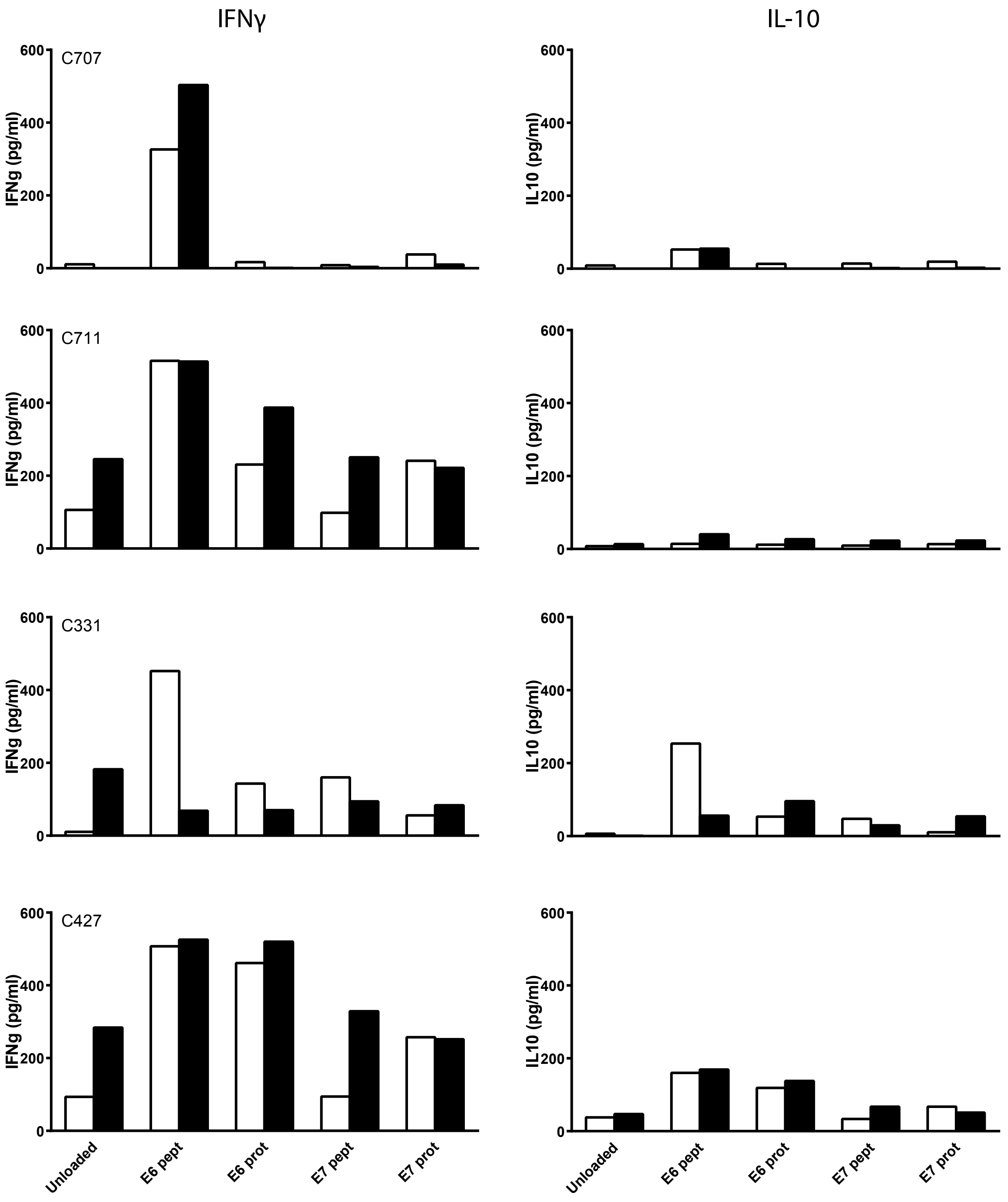

Supplementary Fig. 5 Cytokine production upon secondary stimulation of the LNMC cultures.
LNMC cultures were at day 22 re-stimulated for another round with peptide-loaded autologous monocytes and cultured for another 3 weeks. The functionality and specificity of these re-stimulated bulk cultures were determined by a proliferation assay in which the supernatant of day 2 was used for cytokine analysis by ELISA. Here both the IFNγ and IL-10 production of the first round (white bars) and second round (black bars) of stimulation is given.

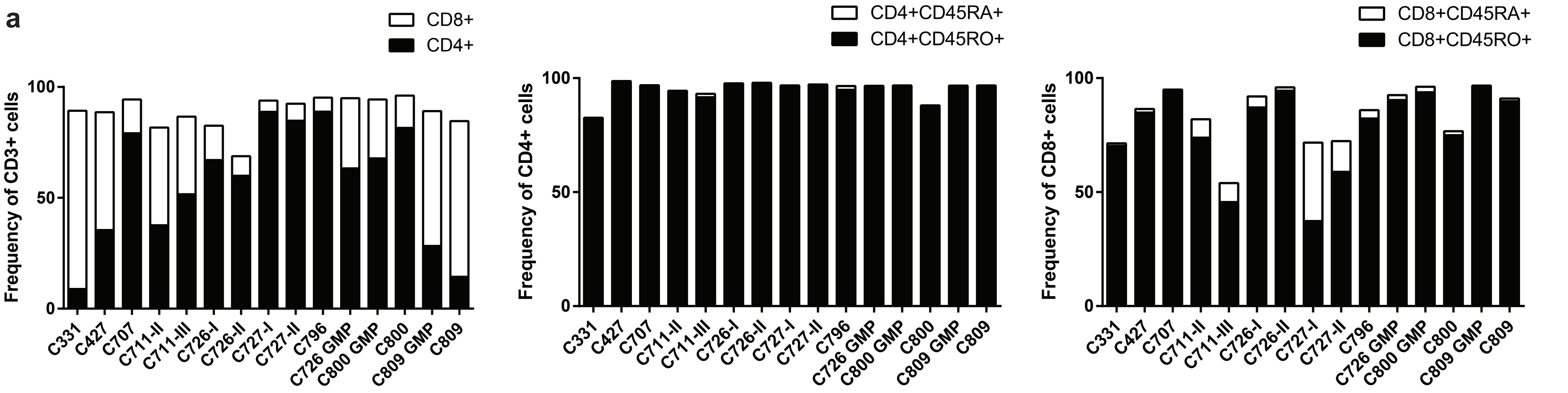

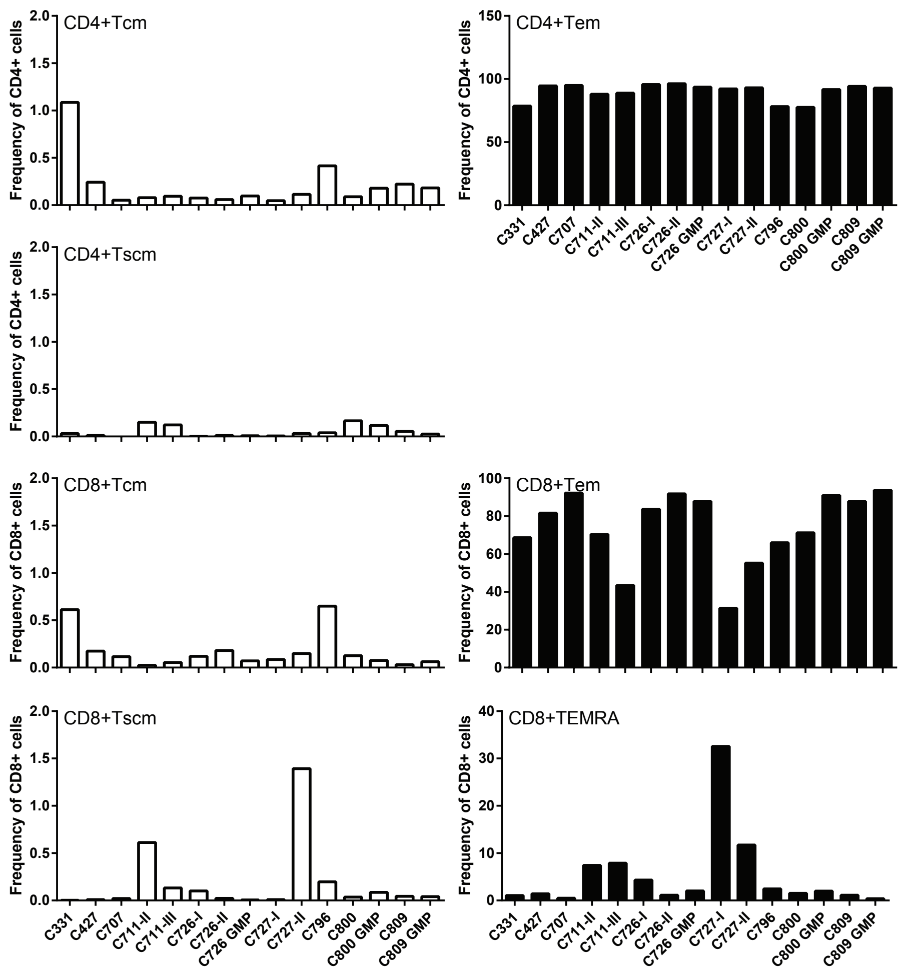

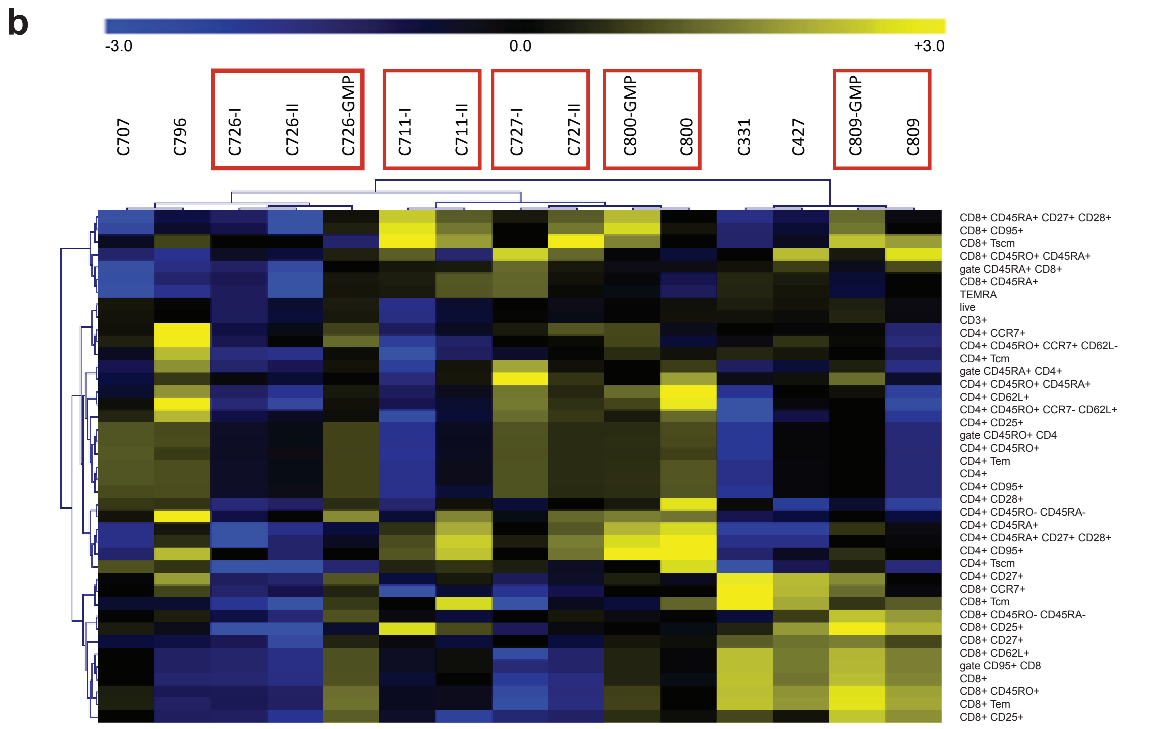

Supplementary Fig. 6 Phenotyping the LNMC for memory markers.
The LNMC were analysed for memory markers by flow cytometry. **a** The frequencies, obtained according to the gating strategy as depicted in Supplementary Fig. 1, are shown for the different subsets of cells (indicated in the top left corner of the graph). **b** This data was subsequently subjected to MeV clustering program to determine markers that are in common (cluster). Tem, effector memory T cells. Tcm, central memory T cells. Tscm, central memory stem cells. TEMRA, effector memory CD45RA+ T cells. The red boxed indicate the replicate bulk cultures that cluster together.

a **b**

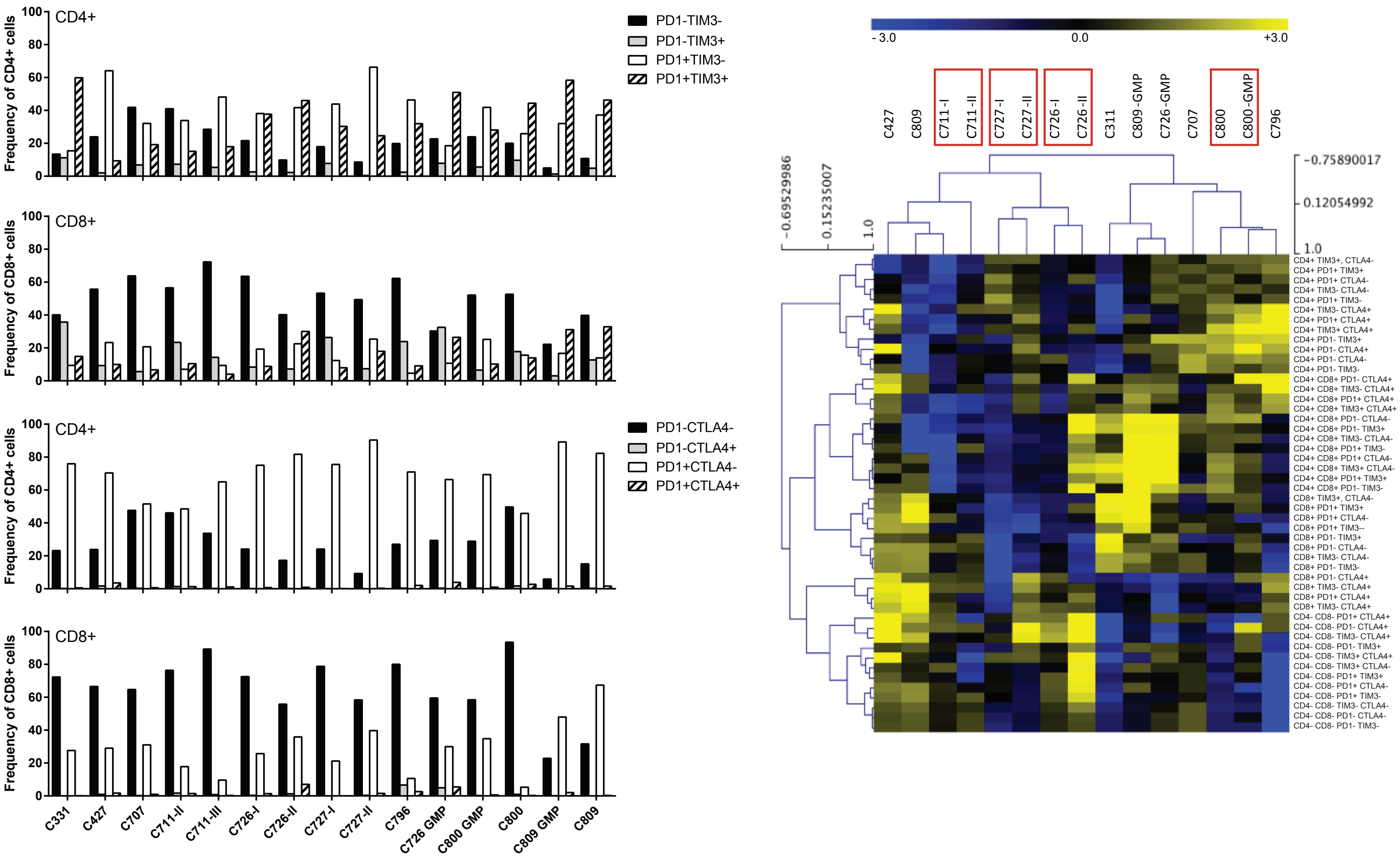

Supplementary Fig. 7 Phenotyping the LNMC for inhibitory markers.

The LNMC were analysed for inhibitory markers by flow cytometry. **a** The frequencies, obtained according to the gating strategy as depicted in Supplementary Fig. 1, are shown for the different subsets of cells (indicated by the legend in the top right corner of the CD4+ graph and is similar for the CD8+_ graph below). **b** This data was subsequently subjected to MeV clustering program to determine markers that are in common (cluster). The red boxed indicate the replicate bulk cultures that cluster together.
